# Detection of plasmid-mediated colistin-resistant and carbapenem-resistant genes by multiplex PCR

**DOI:** 10.1016/j.mex.2018.05.016

**Published:** 2018-05-25

**Authors:** Rujirat Hatrongjit, Anusak Kerdsin, Yukihiro Akeda, Shigeyuki Hamada

**Affiliations:** a Faculty of Science and Engineering, Kasetsart University, Chalermphrakiat Sakon Nakhon Province Campus, Sakon Nakhon, Thailand; b Faculty of Public Health, Kasetsart University, Chalermphrakiat Sakon Nakhon Province Campus, Sakon Nakhon, Thailand; c Department of Infection Control and Prevention, Graduate School of Medicine, Osaka University, Osaka, Japan; d Thailand-Japan Research Collaboration Center on Emerging and Re-emerging Infections, Research Institute for Microbial Diseases, Osaka University, Osaka, Japan

**Keywords:** mPCR for *mcr-1* and carbapenem-resistant genes, PCR, *mcr-1*, *bla*_NDM_, *bla*_KPC_, *bla*_IMP_, *bla*_OXA-48-like_

## Abstract

A multiplex PCR was described to simultaneously detect *mcr-1* and frequently occurring carbapenem-resistant genes including *bla*_KPC_*, bla*_NDM_*, bla*_IMP_, and *bla*_OXA-48-like_ in a single reaction. The PCR product sizes of these 4 carbapenem-resistant genes were 232 bp, 438 bp, 621 bp, and 798 bp for *bla*_IMP_, *bla*_OXA-48-like_, *bla*_NDM_, and *bla*_KPC_, respectively, whereas *mcr-1* revealed 1126 bp of PCR product. This protocol accurately detected those resistant genes in agreement with the reference strains, 127 local carbapenem-resistant Enterobacteriaceae, 8 *mcr-1* carrying Enterobacteriaceae, and 62 carbapenem-susceptible Enterobacteriaceae. This method will be useful for laboratory application and surveillance of carbapenem and/or colistin-resistant bacteria.

## Graphical abstract

**Figure:**
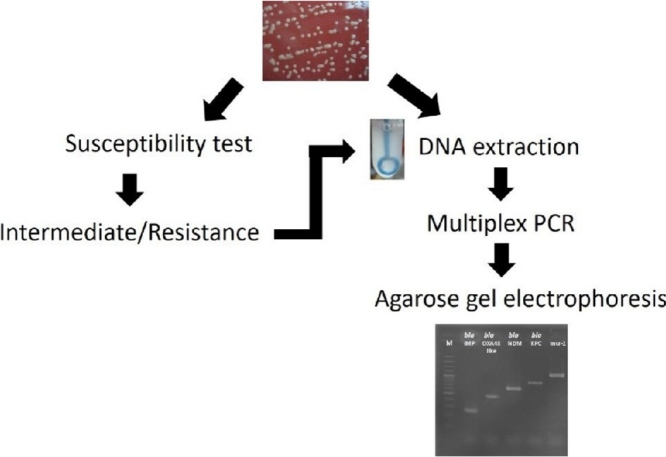

**Specifications Table**

**Table:** 

Subject area	Immunology and Microbiology
More specific subject area	Clinical Bacteriology
Protocol name	mPCR for *mcr-1* and carbapenem-resistant genes
Reagents/tools	1. JumpStart^TM^ REDTaq® ReadyMix^TM^ PCR Reaction Mix (Sigma-Aldrich, USA) 2. Primers IMP-F = 5’-GGAATAGAGTGGCTTAAYTCTC-3’ IMP-R = 5’-GGTTTAAYAAAACAACCACC-3’ OXA48-like-F = 5’-GCGTGGTTAAGGATGAACAC-3’ OXA48-like-R = 5’-CATCAAGTTCAACCCAACCG-3’ NDM-F = 5’-GGTTTGGCGATCTGGTTTTC-3’ NDM-R = 5’-CGGAATGGCTCATCACGATC-3’ KPC-F = 5’-CGTCTAGTTCTGCTGTCTTG-3’ KPC-R = 5’-CTTGTCATCCTTGTTAGGCG-3’ MCR1-F = 5’- GGGTGTGCTACCAAGTTTGC -3’ MCR1-R = 5’- CATTGGCGTGATGCCAGTTT -3’
Experimental design	We modified a multiplex PCR for detection of acquired carbapenemase genes described by Poirel et al. [1] and added the primer to detect *mcr-1* in the same PCR reaction. This method can simultaneously detect 4 prevalent carbapenem-resistant genes (*bla*_IMP_, *bla*_OXA-48-like_, *bla*_NDM_, and *bla*_KPC_) and a colistin-resistant gene (*mcr-1*) in a single reaction and revealed different PCR product sizes that are easy to interpret.
Trial registration	None
Ethics	None

## Value of the protocol

• Simultaneous detection of four frequent clinically relevant carbapenem-resistant genes and *mcr-1* by multiplex PCR in a single reaction.
• Rapid, simple, and reliable for detection of frequently clinically relevant carbapenem and colistin-resistant genes (*mcr-1*) from pure culture.
• Useful for laboratory application and surveillance of carbapenem-resistant and/or colistin-resistant bacteria.
• Useful for detection of isolates co-carry *mcr-1* and carbapenemase genes such as *mcr-1* and *bla*_NDM_.

## Description of protocol

Carbapenem-resistant organisms such as *bla*_KPC_, *bla*_NDM_, *bla*_IMP_, *bla*_OXA48-like_, and the emergence of the *mcr-1* gene, a plasmid-mediated gene that confers colistin resistance in *Enterobacteriaceae,* have both been increasingly recognized worldwide. The spread of *mcr-1*-encoding plasmids into carbapenem-resistant *Enterobacteriaceae* raises concerns about the emergence of untreatable bacteria and it poses a serious threat to public health worldwide.

Many PCR techniques have been described to detect these resistant genes; however, no PCR (especially multiplex PCR) procedure has been described for detecting both *mcr-1* and carbapenem-resistant genes in a single reaction. This study describes a protocol to simultaneously detect *mcr-1* and frequently occurring carbapenem-resistant genes (*bla*_KPC_, *bla*_NDM_, *bla*_IMP_, *bla*_OXA48-like_) as well as to detect co-existence of *mcr-1* and carbapenem-resistant genes in a single reaction from Gram-negative bacteria.

## Major equipment and supplies for PCR assay

• PCR thermal cycler (Takara, Japan or equivalent)
• PCR tubes (Nest Scientific, USA or equivalent)
• Sterile Eppendorf style microcentrifuge tubes (Nest Scientific, USA or equivalent)
• Sterile inoculating loops or needles (Nest Scientific, USA or equivalent)
• Ice bucket or bench top cooler
• Adjustable micropipettors (0.1–1000 μl)
• Aerosol-resistant micropipettor tips (0.1–1000 μl)
• Vortex Mixer (CAPP, Denmark or equivalent)
• Microcentrifuge (CAPP, Denmark or equivalent)

## Reagents for DNA extraction

• Sodium dodecyl sulfate (SDS) (Sigma-Aldrich, USA or equivalent)
• Sodium hydroxide (Sigma-Aldrich, USA or equivalent)

## Reagents for PCR assay

• JumpStart^™^ REDTaq® ReadyMix^™^ PCR Reaction Mix (Sigma-Aldrich, USA)
• PCR grade water (Omega, USA or equivalent)
• 10X Tris-Borate-EDTA buffer (TBE) (Omega, USA or equivalent)
• Agarose gel (Sigma-Aldrich, USA or equivalent)
• Primers (Sigma-Aldrich, USA or equivalent)

## Procedures

### Bacteria and DNA extraction

Bacteria were cultured on blood agar or McConkey agar at 37 °C for 18–24 h. DNA was prepared by heating one or two colonies from an overnight grown plate at 95 °C for 15 min in 30 μl of lysis buffer containing 0.25% (vol/vol) sodium dodecyl sulfate and 0.05 M NaOH. After lysis, 200 μl of sterile distilled water was added to the lysis buffer and the DNA solutions were stored at −20 °C until PCR analysis.

### Multiplex PCR analysis

The multiplex PCR assay was performed in 15-μl reaction mixtures, containing 2 μl of template, 1.5 μl of deionized water, 1X JumpStart^™^ REDTaq® ReadyMix^™^ PCR Reaction Mix (Sigma–Aldrich, USA) and 0.53 μM of each primer (Table 1). The composition of the reagents in the multiplex PCR is shown in the Table 2.

**Table 1:** Primers sequences.

Name	Sequence (5’–3’)	PCR product size (bp)	Reference
IMP-F	GGAATAGAGTGGCTTAAYTCTC	232	[1]
IMP-R	GGTTTAAYAAAACAACCACC		
OXA-48-like-F	GCGTGGTTAAGGATGAACAC	438	[1]
OXA-48-like-R	CATCAAGTTCAACCCAACCG		
NDM-F	GGTTTGGCGATCTGGTTTTC	621	[1]
NDM-R	CGGAATGGCTCATCACGATC		
KPC-F	CGTCTAGTTCTGCTGTCTTG	798	[1]
KPC-R	CTTGTCATCCTTGTTAGGCG		
MCR1-F	GGGTGTGCTACCAAGTTTGC	1126	[2]
MCR1-R	CATTGGCGTGATGCCAGTTT

**Table 2:** Contents of mPCR reaction.

Reagents	Final concentration	μl per reaction
Deionized water	–	1.5
2X JumpStart^TM^ REDTaq® ReadyMix^TM^ PCR reaction mix	1X	7.5
20 μM IMP-F	0.53	0.4
20 μM IMP-R	0.53	0.4
20 μM OXA-48-F	0.53	0.4
20 μM OXA-48-R	0.53	0.4
20 μM NDM-F	0.53	0.4
20 μM NDM-R	0.53	0.4
20 μM KPC-F	0.53	0.4
20 μM KPC-R	0.53	0.4
20 μM MCR1 -F	0.53	0.4
20 μM MCR1 -R	0.53	0.4
DNA extracted	–	2
**Total volume**	–	**15**

The following PCR thermal profile was used: initial activation of DNA polymerase at 95 °C for 3 min; 30 cycles of denaturation at 95 °C for 30 s, primer annealing at 56 °C for 30 s and extension at 72 °C for 45 s, and a final extension at 72 °C for 5 min. The PCR products were analyzed using gel electrophoresis for 30 min on 2% agarose gels in 0.5X TBE buffer. The gels were stained with ethidium bromide and visualized under ultraviolet light (GeneGenius Bioimaging System, SynGene). The sizes of the PCR products were determined by comparison with a molecular-sized standard (GeneRuler™ 100 bp Plus DNA ladder, Thermo Fisher Scientific).

### Interpretation

**Fig. 1:**
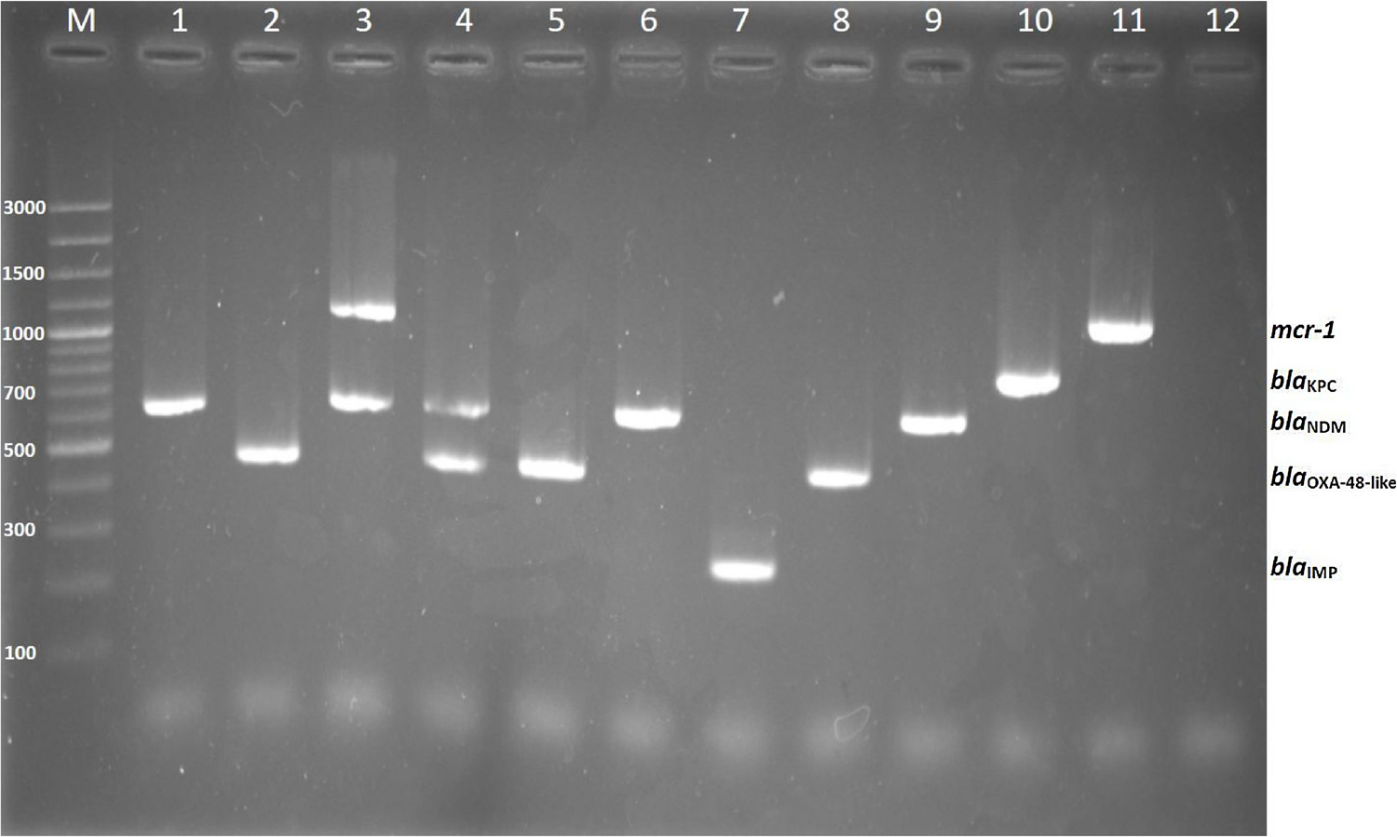
Agarose gel electrophoresis of PCR-amplified products from the representative four carbapenem-resistant genes and *mcr-1*. Lane M = 100 bp DNA ladder, lane 1 = *K. pneumoniae* strain no.1385 (*bla*_NDM_), lane 2 = *K. pneumoniae* strain no. 1386 (*bla*_OXA-48-like_), lane 3 = *E. coli* strain no.A434-59 (*bla*_NDM_ and *mcr-1*), lane 4 = *E. coli* strain no.98 (*bla*_NDM_ and *bla*_OXA-48-like_), lane 5 = *E. coli* strain no.1387 (*bla*_OXA-48-like_), lane 6 = *K. pneumoniae* strain no.1263 (*bla*_NDM_), lane 7 = *K. pneumoniae* strain 22 (*bla*_IMP-14a_), lane 8 = *K. pneumoniae* ATCC® BAA-2524^™^ (*bla*_OXA48_), lane 9 = *E. coli* ATCC® BAA-2452^™^ (*bla*_NDM-1_), lane 10 = *K. pneumoniae* ATCC® BAA-1705^™^ (*bla*_KPC_), lane 11 = *E. coli* AK1 (a strain carrying *mcr-1* recombinant plasmid), and lane 12 = negative control (distilled water).

As shown in Fig. 1, our multiplex PCR differentiated 4 prevalent carbapenemase genes and *mcr-1*. The PCR product sizes of these 4 carbapenemase genes were about 232 bp, 438 bp, 621 bp, and 798 bp for *bla*_IMP_, *bla*_OXA-48-like_, *bla*_NDM_, and *bla*_KPC_, respectively, whereas *mcr-1* revealed 1126 bp of PCR product.

### Validation

We have validated our protocol with reference strains including *Klebsiella pneumoniae* ATCC® BAA-1705^™^ (*bla*_KPC_), *Escherichia coli* ATCC® BAA-2340^™^ (*bla*_KPC_), *E. coli* ATCC® BAA-2452^™^ (*bla*_NDM-1_), *E. coli* ATCC® BAA-2469^™^ (*bla*_NDM-1_), *K. pneumoniae* ATCC® BAA-2524^™^ (*bla*_OXA-48_), Serratia marcescens KU3838 (*bla*_IMP-6_), *K. pneumoniae* strain 22 and strain 34 (*bla*_IMP-14a_) [3], and *E. coli* A434-59 (*mcr-1* and *bla*_NDM-1_) [2]. The multiplex PCR revealed PCR product sizes of 232 bp for *bla*_IMP-6_ and *bla*_IMP-14a_ in *S. marcescens* KU3838 and *K. pneumoniae* strain 22 and 34, 438 bp for *bla*_OXA-48_ in *K. pneumoniae* ATCC® BAA-2524^™^, 621 bp for *bla*_NDM_ in *E. coli* ATCC® BAA-2452^™^, *E. coli* ATCC® BAA-2469^™^and *E. coli* A434-59, 798 bp for *bla*_KPC_ in *K. pneumoniae* ATCC® BAA-1705^™^ and *E. coli* ATCC® BAA-2340^™^, and 1126 bp for *mcr-1* in *E. coli* A434-59, respectively.

In total, testing with our multiplex PCR was undertaken on 127 carbapenem-resistant *Enterobacteriaceae* with known carbapenemase genes using either Sanger sequencing or next-generation sequencing. These 127 isolates consisted of 50 *bla*_NDM-1_ harboring isolates, 19 *bla*_NDM-5_ harboring isolates, 1 *bla*_NDM-7_ harboring isolates, 32 *bla*_OXA-181_ harboring isolates, 10 *bla*_OXA-232_ harboring isolates, 5 isolates carrying *bla*_NDM-1_ and *bla*_OXA-181_, 4 isolates carrying *bla*_NDM-1_ and *bla*_OXA-232_, and 6 *bla*_IMP14_ harboring isolates, respectively. As expected, this multiplex PCR assay could detect these carbapenemase genes in agreement with either Sanger sequencing or next-generation sequencing results e.g., isolates containing *bla*_OXA-181_ and *bla*_OXA-232_ revealed 438 bp of *bla*_OXA-48-like_ PCR product, while isolates carrying *bla*_NDM-1_, *bla*_NDM-5_ and *bla*_NDM-7_ showed about 621 bp of *bla*_NDM_ PCR product. Where isolates contained either *bla*_NDM-1_ and *bla*_OXA-181_ or *bla*_NDM-1_ and *bla*_OXA-232_, our PCR revealed 2 bands at 438 bp and 621 bp for *bla*_OXA-48-like_ and *bla*_NDM_, respectively (Fig. 1).

We also tested the multiplex PCR with 8 known *mcr-1* isolates using Sanger sequencing (6 *E. coli* and 2 *K. pneumoniae*). The PCR assay could accurately detect the 1126 bp of *mcr-1* in 8 isolates. In addition, *E. coli* A434-59 a strain co-carry of *mcr-1* and *bla*_NDM-1_ [2] revealed 2 bands of 1126 bp (*mcr-1*) and 621 bp (*bla*_NDM-1_) by this PCR (Fig. 1). Sixty-two carbapenem-susceptible *Enterobacteriaceae* were tested using the multiplex PCR and the results revealed no PCR product bands.

This method has advance in case of easy to use and save cost and time to simultaneously detect 4 frequently occurring carbapenemase genes and *mcr-1* in a single reaction.
